# Disentangling the Dyadic Dance: Theoretical, Methodological and Outcomes Systematic Review of Mother-Infant Dyadic Processes

**DOI:** 10.3389/fpsyg.2018.00348

**Published:** 2018-03-19

**Authors:** Livio Provenzi, Giunia Scotto di Minico, Lorenzo Giusti, Elena Guida, Mitho Müller

**Affiliations:** ^1^Center for the at-Risk Infant, Scientific Institute IRCCS Eugenio Medea, Bosisio Parini, Italy; ^2^Department of Psychology, Ludwig-Maximilians-University Munich, Munich, Germany

**Keywords:** attunement, contingency, matching, mother-infant dyad, mutuality, reciprocity, reparation, synchrony

## Abstract

**Background:** During the last decades, the research on mother-infant dyad has produced a great amount of data, methods and theories, which largely contributed to set a revolution in the way we look at developmental changes during infancy and childhood. Very different constructs depict the different aspects of the “dyadic dance” occurring between a mother and her infant; nonetheless, a comprehensive and consistent systematization of these concepts in a coherent theoretical landscape is still lacking.

**Aim:** In the present work, we aim at disentangling the different theoretical and methodological definitions of 9 dyadic constructs and we highlight their effects on infants' and children developmental outcomes.

**Methods:** A literature search has been conducted on three databases—PubMed, Scopus, Web of Science. Three different reviews are reported here: (1) a review on the theoretical definitions of dyadic constructs; (2) a review of operational definitions, settings and methods of dyadic processes; (3) a systematic review of dyadic processes' outcomes for infants' and children developmental trajectories.

**Results:** Two constructs emerged as wide meta-theoretical concepts (reciprocity and mutuality) and seven described specific processes (attunement, contingency, coordination, matching, mirroring, reparation, synchrony). A global model resuming the relationships among different processes is reported, which highlights the emergence of two specific cycles of dyadic functioning (i.e., matching-mismatching-reparation-synchrony; contingency, coordination, attunement, mirroring). A comprehensive review of the adopted measures is also provided. Finally, all the processes provided significant contributions to infants' behavioral, cognitive, and socio-emotional development during the first 3 years of age, but limited research has been conducted on specific processes (e.g. reparation and mirroring).

**Conclusion:** The present study provides an original research-grounded framework to consider the different nature of mother-infant dyadic processes within a unified dyadic eco-system. Different levels of evidence emerged for the role of diverse mother-infant dyadic processes on infants' and children development. Open questions and future research directions are highlighted.

## Introduction

### At the origins of mother-infant dyadic processes research

Compared to other mammalian species, human newborns present larger and more adaptable brains that are also particularly immature and dependent on caregiving behaviors and environment (Trevathan, 2015). Not only is human infants' survival and safety granted by the caregiving environment, but the developmental outcomes themselves are shaped by the early context of care and caregiver-infant interaction (O'Connor, 2003; Swain et al., 2007). Thus, human caregivers—more often the mothers—have to respond to infant basic needs (such as feeding, sleep, and temperature regulation), and also play a crucial role in fostering regulative (Conradt and Ablow, 2010), emotional (Provenzi et al., 2015a), cognitive (Malmberg et al., 2016) and social abilities (Kivijärvi et al., 2001), that form developmental key competencies for later life adaptation and both physical and psychological health. Consequently, the multilayered and complex interactional processes occurring between infants and their mothers is of vital importance for healthy developmental trajectories.

The scientific interest in the form, quality and developmental relevance of early caregiver-infant relationship is as old as Bowlby's trilogy on attachment (Bowlby, 1969/1982, 1973, 1980). Based on these works, Ainsworth and colleagues (Ainsworth et al., 1978) considered maternal sensitivity, i.e., the ability to perceive infant signals, to interpret them correctly and to promptly and appropriately respond to them, as the key component in mother-infant relationship. While this concept has been critical to understand infant attachment (Behrens et al., 2016), it was recognized as a global, unidirectional scale that does not help in depicting the underlying mechanisms which occur moment-by-moment and require bidirectional or reciprocal contributions of both the mother and the infant behavioral and emotional states (Mesman, 2010).

Stern (1971) was among those who pioneered the idea that mother and infant engage in a bidirectional moment-to-moment process, in which, together, both partners form repeated dyadic patterns of body-movements and gaze-behaviors—a view that emphasized infant readiness for social encounters and social-based meaning making. Consequently, the interest in infants' communicative abilities, dyadic exchanges and early forms of intersubjectivity rapidly grew during the 70s and 80s (e.g., Sander, 1977; Brazelton, 1979; Trevarthen, 1979, 1980; Stern, 1985). Bateson (1979) described the interaction between caregiver and infant as eye-contacts and vocalizations forming a “proto-conversation” primarily characterized by mutuality. Others highlighted the active role played by infants in early face-to-face interactions proposing that the trend and drive to intersubjectivity is something innate at birth in human infants (e.g., Murray and Trevarthen, 1986). Infants were increasingly viewed as competent social partners, who anticipate the caregivers' responses to their own communicative signals and react with, for example, withdrawal or protest to the caregiver's social disengagement (e.g., the Still-Face paradigm; Brazelton et al., 1975; Papoušek and Papoušek, 1977; Tronick et al., 1978).

Tronick was among the first to provide a formalized and consistent theoretical model of the reciprocal nature of caregiver-infant-interactions called the Mutual-Regulation-Model (Gianino and Tronick, 1988). This model was mainly grounded in the growing assumption supported by the infant research field, which highlighted that the caregiver-infant-dyad forms a mutually coordinated, communicative unit that quickly oscillates between synchronous states of affective-behavioral matches and asynchronous states of affective-behavioral mismatches in a continuous moment-to-moment process of mutual behavioral adaption, emotional exchange and affect regulation.

To date, the view on the caregiver-infant dyad has been expanded by non-linear dynamic system approaches (Hollenstein, 2007; DiCorcia and Tronick, 2011; Sravish et al., 2013; Beebe et al., 2016). Consistent with this framework, the caregiver-infant-dyad is viewed as an interactional system, in which both partners hierarchically organize levels of functioning (behavioral, affective, physiological, etc.) by reciprocally and mutually coordinating their behaviors, communicative signals and emotional states in various domains, in which changes on one level affect the functioning (and development) on others.

### Mother-infant interactions: what happens in-between matters

The nature of the reciprocal interconnections and interactions between a mother and her infant is complex and multifaceted and what takes place between the two interactive partners is made up of multiple processes. To understand these dyadic mechanisms, research started to analyze the micro-temporal features of these social exchange processes. In their review, Leclère et al. (2014) focused on the dyadic concept of “synchrony” and defined it as the intermodal temporal coordination of verbal and non-verbal communicative and emotional behaviors between interactive partners. They confirmed that synchrony is higher between familiar, healthy partners and is associated with more positive child outcomes. However, they recognized that various terms (mutuality, reciprocity, rhythmicity, etc.) and assessment methods (global scales, specific synchrony scales, micro-temporal time series analyses) have been used to describe and measure dyadic synchrony.

This paucity of conceptual clarity doesn't only apply to the concept of synchrony, but also to other dyadic concepts that have been proposed by diverse authors and throughout scientific progress in the infant research field. Stern (2010) dedicated more attention to affective attunement between mother and infant, i.e., the maternal matching and modulation of the infant affective tone. Sander (1988) addressed the importance of mutual event-structure in moment-to-moment interactions between caregivers and infants on neonatal state organization and biological regulation. Beebe and Lachmann (1998) captured Fogel's (1992) concept of co-regulation (i.e., a concept denoting individuals' behaviors which continuously modify and shape each other) and later understood self- and interactive contingency as the temporal relation between the occurrence of affective-behavioral events, that involves sequential coordination (Beebe et al., 2016). Fonagy and Target (2006) see the caregivers' attuned, contingent and empathic mirroring responses to infant's emotional signals as the key element in the acquisition of emotion regulation, whereas they consider “marked” mirroring (i.e., exaggerated modulations of infant signals) as the optimal mode for infants to internalize the expression of their own feelings. Again, Tronick stated that most dyadic interaction processes are “messy” in the sense that they normally feature naturally occurring mismatched states, but the successful reparation of these dyadic mismatches is highly important for the development of infant regulation, resilience and of other domains (DiCorcia and Tronick, 2011). Thus, perfect synchrony between the temporal sequences of each partners' behaviors might be neither desirable nor possible, nor can the process of interactive reparation be sufficiently measurable by assessments of synchrony.

First, it is not clear if these theoretical constructs are meant to be actual interactive processes describing what happens in the dyad in a moment-by-moment fashion or rather if they are broader meta-theoretical accounts of mother-infant dyads. Notably, there is a lack of systematization regarding which theoretical items are concepts (i.e., meta-theoretical views of mother-infant dyadic interactions) and which of them are processes (i.e., detailed descriptions of specific joint actions observable within the mother-infant dyad). Moreover, the relationships among these dyadic concepts has not been previously accounted for. It is our belief, that all these dyadic constructs reflect specific facets of the complex micro-temporal dyadic nature of the mother-infant system. Additionally, there is also a lack of agreement and systematization at the methodological level. For instance, interactive reparation might be alternatively measured as the frequency of transitions from mismatched to matched states (e.g., Provenzi et al., 2015b) or as the average mismatch duration (e.g., Müller et al., 2015), while synchrony is usually assessed by time-series-analyses and lead-lag-relationships (e.g., Feldman, 2003).

### The need of a dyadic dictionary and roadmap

In the previous paragraphs a very brief sketch on previous infant research decades has been laid out in order to highlight that when it comes to measuring and understanding what happens within the mother-infant dyad in research, different terms are often used interchangeably. This leads to an overall confusion in both theoretical and methodological level. Consistently, it is plausible that this theoretical and methodological disarray of infant research might have led to mixed findings in previous research. After more than four decades of studies in the field, we believe it is time to provide a first attempt to systemize not only the evidence, but also the theoretical underpinnings and the methodologies used in this area of investigation. On the one hand, it might be useful to develop a unified dyadic dictionary in which each term could have a theoretical definition based on previous contributions in literature and in which the actual and potential relationships among different dyadic processes are highlighted or, at least, hypothesized. On the other hand, a methodological roadmap is also needed to navigate the multifaceted landscape of methods that have been previously used to quantify mother-infant interactive states and behaviors. Finally, this comprehensive systemization also holds the potentials of highlighting what previous decades of infant research tradition has found about the protective and risk factors inherent to adaptive and maladaptive caregiver-infant environments.

### Aims

In the present work, we aimed to disentangle the different dyadic constructs (i.e., both concepts and processes) which can be depicted and measured when observing mother-infant interacting dyads. Differences and similarities among different dyadic constructs will be highlighted through a three-step systematic review. First, we will review the theoretical definition of dyadic constructs, according to a qualitative approach plus a computer-aided text-analysis of the included papers (section: Theoretical Review). Second, methodological aspects will be abstracted and reviewed, in order to provide a systematic overview of the procedures adopted in previous literature to measure specific dyadic processes in the mother-infant interaction (section: Methodological Review). Third, findings of previous studies will be reviewed describing specific characteristics of dyadic processes, their predictors and effects on infants and children development, as well as differences among low- and high-risk mother-infant dyads (section: Systematic Review of Outcomes).

## Methods

### Data selection

**Figure 1:**
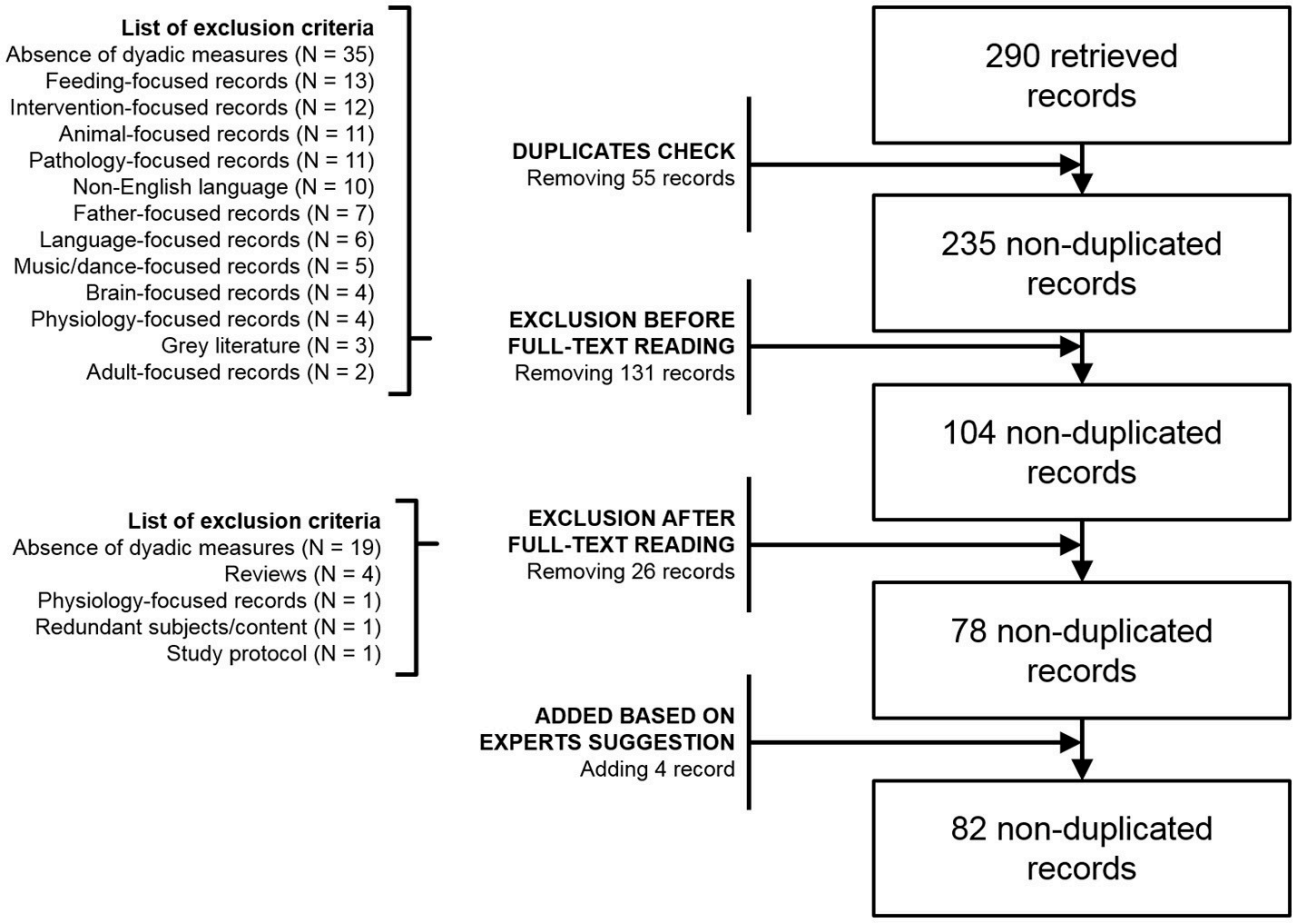
Flow chart of literature search and study selection.

The procedures of the present study are consistent with the guidelines for systematic review included in the Referred Reporting items for Systematic Review and Meta-Analysis (PRISMA) guidelines (Liberati et al., 2009). The literature search was performed on three different databases: Scopus, PubMed and Web of Science. The search string included: “mother” AND “infant” AND (“attunement” OR “contingency” OR “coordination” OR “matching” OR “mirroring” OR “mutuality” OR “reciprocity” OR “reparation” OR “synchrony”). The records were checked for duplicates using Mendeley 1.17.6 (© 2008–2016 Mendeley Ltd.). The remaining papers were than screened independently by three authors (LP, MM, and LG) by reading titles, abstracts and full-texts. Disagreements were solved in conference. Exclusion criteria and the whole selection process are reported in the flow chart in Figure 1.

### Data abstracting

The records were reviewed and the following data was extracted: authors, year, journal source, dyadic construct(s) included, theoretical definition, operation definition, measurement and instrument/tool, sample characteristics, sample size, setting, observational procedure, findings (separated in four categories: characterization, antecedents, consequences and between-group differences for the included dyadic process). According to the PICOS questions for systematic review, population was mother-infant dyads, comparators were sample characteristics, setting, type of observational procedure, outcomes were different effects of dyadic processes on maternal and infants' domains, whereas no exclusion criterion was applied to study designs (but see quality appraisal below). Interventions were not included in the present review. The final pool of included studies is reported in Table 1.

**Table 1:** Characteristics of the included studies.

**Dyadic concept/process**	**Paper**	**Journal**	**Sample size**	**Infant characteristics**	**Mother characteristics**	**Environment**	**Procedure**
**ATTUNEMENT**							
	Feldman and Greenbaum, 1997	Infant Mental Health Journal	36	3.5 months; healthy	Healthy	Lab	Free FTF interaction
	Jonsson et al., 2001	Scandinavian Journal of Psychology	39	2-to-12 months; healthy	Healthy	Home	Free FTF interaction
	Jonsson and Clinton, 2006	Infant and Child Development	27	2-to-12 months; healthy	Healthy	Home	Free FTF interaction
	Leyendecker et al., 1997	Infant Behavior and Development	41	3.5 months; healthy	Immigrants	Home	Routine care
	Nicely et al., 1999b	Infant Behavior and Development	77	9-to-13 months; healthy	Healthy	Home	Structured FTF interaction
**CONTINGENCY**							
	Beebe et al., 2010	Attachment and Human Development	84	4 months; healthy	Healthy	Lab	Free FTF interaction
	Beebe et al., 2011	Infant Mental Health Journal	119	4 months; healthy	Healthy	Lab	Free FTF interaction
	Beebe et al., 2012a	Psychoanalytic Dialogues	84	4 months; disorganized attachment	Healthy	Lab	Free FTF interaction
	Beebe et al., 2012b	Psychoanalytic Psychology	132	4 months; healthy	Depressed	Lab	Free FTF interaction
	Beebe et al., 2016	Developmental Psychology	132	4 months; healthy	Healthy	Lab	Free FTF interaction
	Bigelow and Rochat, 2006	Infancy	29	2 months; healthy	Healthy	Lab	Free FTF interaction
	Braarud and Stormark, 2006	Infant and Child Development	45	3 months; healthy	Healthy	Lab	Replay paradigm
	Braarud and Stormark, 2008	Social Development	32	4 months; healthy	Healthy	Lab	Replay paradigm
	Brighi, 1997	Infant and Child Development	48	6 months; healthy	Healthy	Lab	Structured FTF interaction
	Cohn and Elmore, 1988	Infant Behavior and Development	20	3 months; healthy	Healthy	Lab	Free FTF interaction
	Cote et al., 2008	Infancy	121	5 months; healthy	Immigrants	Home	Routine care
	Crown et al., 1996	Journal of Psycholinguistic Research	53	1.5-to-12 months; healthy	Healthy	Lab	Free FTF interaction
	Kärtner et al., 2010	Child Development	44	1-to-3 months; healthy	European + African	Home	Free FTF interaction
	Lohaus et al., 2005	Journal of Genetic Psychology	87	3 months; healthy	Healthy	Home	Structured FTF interaction
	Malatesta et al., 1989	Monographs of the Society for Research in Child Development	58	2-to-22 months; healthy	Healthy	Lab	Separation paradigm
	Mendes and Seidl-de-Moura, 2014	Spanish Journal of Psychology	60	1-to-5 months; healthy	Healthy	Home	Free FTF interaction
	Murray et al., 2016	Scientific Reports	91	0.5-to-2.5 months; healthy	Healthy	Home	Free FTF interaction
	Pomerleau et al., 2003	Infant Mental Health Journal	68	1-to-6 months; healthy	Adult + teen-age	Home	Structured FTF interaction
	Striano et al., 2006	Interaction Studies	33	3-to-6 months; healthy	Healthy	Lab	Replay paradigm
	Suwalsky et al., 2012	Infant Behavior and Development	62	5.5 months; adopted	Healthy	Home	Free FTF interaction
**COORDINATION**							
	Crown et al., 2002	Journal of Psycholinguistic Research	45	1.5 months; healthy	Healthy	Lab	Free FTF interaction
	De Barbaro et al., 2013	Human Development	5	4-to-12 months; healthy	Healthy	Home	Structured FTF interaction
	Feldstein et al., 1993	Infant Behavior and Development	28	4 months; healthy	Healthy	Lab	Free FTF interaction
	Hammal et al., 2015	IEEE Transactions on Affective Computing	42	4 months; healthy	Healthy	Lab	FFSF procedure
	Hane et al., 2003	Journal of Psycholinguistic Research	34	4 months; healthy	Healthy	Lab	Routine care
	Harder et al., 2015	Developmental Psychology	41	4-to-10 months; healthy	Healthy	Lab	Free FTF interaction
	Kokkinaki et al., 2017	Infant and Child Development	11	2-to-6 months; healthy	Healthy	Home	Free FTF interaction
	Montirosso et al., 2010	British Journal of Developmental Psychology	50	6-to-9 months; preterm	Healthy	Lab	FFSF procedure
	Moore and Calkins, 2004	Developmental Psychology	73	3 months; healthy	Depressed	Lab	FFSF procedure
	Northrup and Iverson, 2015	Infancy	35	9 months; siblings of children with ASD	Healthy	Home	Structured FTF interaction
	Provenzi et al., 2015b	Infant Behavior and Development	40	4 months; healthy	Healthy	Lab	FFSF procedure
	Rutter and Durkin, 1987	Developmental Psychology	18	12-to-24 months; healthy	Healthy	Lab	Free FTF interaction
	Zlochower and Cohn, 1996	Infant Behavior and Development	35	4 months; healthy	Depressed	Lab	Free FTF interaction
**MATCHING**							
	Deckner et al., 2003	Infancy	30	18 and 24 months; healthy	Healthy	Lab	Structured FTF interaction
	Field et al., 1990	Developmental Psychology	48	3 months; healthy	Depressed, low SES	Lab	Free FTF interaction
	Montirosso et al., 2015	Infant Behavior and Development	75	4 months; healthy	Healthy	Lab	FFSF procedure
	Moore and Calkins, 2004	Developmental Psychology	73	3 months; healthy	Depressed	Lab	FFSF procedure
	Nicely et al., 1999a	Infant Behavior and Development	38	11-to-13 months; healthy	Healthy	Home	Structured FTF interaction
	Nicely et al., 1999b	Infant Behavior and Development	77	9-to-13 months; healthy	Healthy	Home	Structured FTF interaction
	Noe et al., 2015	Psychopathology	68	3.5 months; healthy	Depressed	Lab	FFSF procedure
	Reck et al., 2011	Infant Mental Health Journal	62	1-to-8 months; healthy	Depressed	Lab	FFSF procedure
	Riva Crugnola et al., 2014	Infant Behavior and Development	60	3 months; healthy	Adolescent mothers	Lab	Free FTF
	Riva Crugnola et al., 2016	Psychopathology	71	3 months; healthy	Depressed, anxious, distressed	Lab	Free FTF
	Tronick and Cohn, 1989	Child development	54	3-to-9 months; healthy	Healthy	Lab	FFSF procedure
	Weinberg et al., 2006	Journal of Child Psychology and Psychiatry and Allied Disciplines	133	3 months; healthy	Depressed	Lab	FFSF procedure
**MIRRORING**							
	Bigelow and Walden, 2009	Infancy	38	4 months; healthy	Healthy	Lab	FFSF procedure
	Kim et al., 2014	Infant Behavior and Development	50	7 months; healthy	Healthy	Lab	Free FTF
	Lavelli and Fogel, 2013	Developmental Psychology	24 dyads	1-3 months; healthy	Healthy	home	Free FTF interaction
	Murray et al., 2016	Scientific Reports	91	0.5-to-2.5 months; healthy	Healthy	home	Free FTF interaction
	Sarfi et al., 2011	Infant Behavior and Development	71	6 months; healthy	Methadone during pregnancy	lab	Free FTF interaction
**MUTUALITY**							
	Savonlahti et al., 2005	Nordic Journal of Psychiatry	26	6 months; healthy	Substance-dependent	Lab	Routine care
	Van Egeren et al., 2001	Developmental psychology	150	4 months; healthy	Healthy	Home	Free FTF interaction
	White-Traut et al., 2013	Infant Behavior and Development	198	1.5 months; preterm	Minority status, low SES	Home	Routine care
**RECIPROCITY**							
	Feldman, 2010	Attachment and Human Development	36	3.5 months; healthy	Healthy	Lab + home	Structured FTF interaction
	Ferber and Feldman, 2005	Infancy	81	1.5 months; healthy	Depressed/anxious	Home	Free FTF interaction
	Lowinger, 1999	Infant and Child Development	56	2.5 months; healthy	Healthy	Home	Structured FTF interaction
	Mayes et al., 1997	Infant Behavior and Development	81	3-to-6 months; healthy	Substance-dependent	Lab	Free FTF interaction
	Roe and Drivas, 1997	American Journal of Orthopsychiatry	147	3 months; preterm	Healthy	Home	Free FTF interaction
**REPARATION**							
	Montirosso et al., 2015	Infant Behavior and Development	75	4 months; healthy	Healthy	Lab	FFSF procedure
	Müller et al., 2015	Psychopathology	46	3-to-4 months; healthy	Anxious	Lab	FFSF procedure
	Provenzi et al., 2015b	Journal of Experimental Child Psychology	65	4 months; healthy	Healthy	Lab	FFSF procedure
	Reck et al., 2011	Infant Mental Health Journal	62	1-to-8 months; healthy	Depressed	Lab	FFSF procedure
**SYNCHRONY**							
	Bernieri et al., 1988	Journal of Personality and Social Psychology	8	14-to-18 months; healthy	Healthy	Lab	Structured FTF interaction
	de Graag et al., 2012	Infant Behavior and Development	84	5 months; healthy	Healthy	Lab	FFSF procedure
	Doi et al., 2011	Journal of Physiological Sciences	48	10 months; healthy	Healthy	Home	Routine care
	Dowd and Tronick, 1986	Child development	4	0.5 months; healthy	Healthy	Lab	Free FTF interaction
	Feldman et al., 1996	Journal of Applied Developmental Psychology	36	3.5 months; healthy	Healthy	Lab	Free FTF interaction
	Feldman and Greenbaum, 1997	Infant Mental Health Journal	36	3.5 months; healthy	Healthy	Lab	Free FTF interaction
	Feldman, 2006	Developmental Psychology	71	3 months; extremely and moderate preterm	Healthy	Home	Free FTF interaction
	Feldman et al., 2011	Infant Behavior and Development	40	3 months; healthy	Healthy	Lab	Free FTF interaction
	Field et al., 1989	Infant Behavior and Development	16	3 months; healthy	Depressed	Lab	Free FTF interaction
	Field et al., 1990	Developmental Psychology	48	3 months; healthy	Depressed, low SES	Lab	Free FTF interaction
	Granat et al., 2017	Emotion	100	9 months; healthy	Depressed/ anxious	Home	Free FTF interaction
	Gratier, 2003	Cognitive Development	60	2-to-5 months; healthy	Immigrants	Home	Free FTF interaction
	Ham and Tronick, 2009	Psychotherapy Research	18	5 months; healthy	Healthy	Lab	FFSF procedure
	Hammal et al., 2015	IEEE Transactions on Affective Computing	42	4 months; healthy	Healthy	Lab	FFSF procedure
	Harel et al., 2011	Infancy	60	3 months; preterm	Healthy	Home	Free FTF interaction
	Kaitz et al., 2010	Infant Behavior and Development	93	6 months; healthy	Anxious	Lab	FFSF procedure
	Karger, 1979	Child development	49	1-to-3 months; preterm	Healthy	Lab + home	Routine care
	Leclère et al., 2016	Transl Psychiatry	20	12-to-36 months; atypical development	Healthy	Lab	Free FTF interaction
	Lester et al., 1985	Child development	40	3-to-5 months; preterm	Healthy	Lab	Free FTF interaction
	Lotzin et al., 2015	PLoS ONE	68	4-to-9 months; healthy	Depressed	Lab	FFSF procedure
	Lotzin et al., 2016	Development and Psychopathology	68	4-to-9 months; healthy	Depressed	Lab	FFSF procedure
	Moore and Calkins, 2004	Developmental Psychology	73	3 months; healthy	Healthy	Lab	FFSF procedure
	Moore et al., 2016	Infant Behavior and Development	75	7 months; healthy	Depressed / anxious	Lab	FFSF procedure
	Tronick and Cohn, 1989	Child development	54	3-to-9 months; healthy	Healthy	Lab	FFSF procedure
	Van Puyvelde et al., 2010	Infant Behavior and Development	15	3 months; healthy	Healthy	Lab	Free FTF interaction
	Weinberg et al., 2006	Journal of Child Psychology and Psychiatry and Allied Disciplines	133	3 months; healthy	Healthy	Lab	FFSF procedure

### Quality appraisal

The methodological quality of the included papers was assessed according to the Quality Assessment Tool for Quantitative Studies (Jackson et al., 2005). Sections A-F (A, selection bias; B, study design; C, confounders; D, blinding; E, data collection methods; F, withdrawal and dropouts) were coded by three independent researchers (LP, GSM, EG) as 3 (weak), 2 (moderate) or 1 (strong) according to the component rating scale criteria. A summary 1-to-3 score was assigned to each paper according to the presence of 2 or more weak scores (3, weak), only 1 weak score (2, moderate), no weak scores (1, strong). A 93% agreement was reached for the A-F components. Disagreement was solved in conference through the supervision of the third author.

### Data synthesis and analysis

#### Theoretical review

The review of theoretical definitions of dyadic concepts and processes was done in two sub-steps: a human-driven analysis of theoretical definitions and a computer-aided text-analysis of the included papers' introduction sections.

First, theoretical definitions have been abstracted from the introduction sections of the included papers. They have been isolated and saved in a word processing file, separately for each dyadic process (i.e., attunement, contingency, coordination, matching, mirroring, mutuality, reciprocity, reparation, synchrony). The reported theoretical definitions have been screened and keywords in the definitions have been highlighted in different colors (e.g., time-related words highlighted in red; body-part involved highlighted in green; cognitive processes highlighted in yellow; emotion-related words highlighted in blue; etc.). Keywords highlighted in the same colors have been merged together in order to obtain separate theoretical definitions that included the overlapping keywords among different papers and which excluded non-overlapping ones.

Second, the introduction of the included papers were saved as.txt files and were elaborated using the text-analysis software T-LAB (Lancia, 2015). T-LAB is a text-mining software developed to detect meaningful relationships between words or sub-sections of a given text (Provenzi et al., 2016). This software encompasses a set of linguistic and statistical tools for content analysis and text mining. The imported.txt files have been analyzed using the word-sequence tool of T-LAB, which is based on probability analysis of word co-occurrence and allows to obtain an assessment of the antecedents and successors of target keywords (i.e., the dyadic processes in the present text) (for a full description of the analysis, please refer to Lancia, 2015, http://bit.ly/2ryYfMS). Based on word co-occurrence analysis, T-LAB provides the odds of the associations between selected words (in this case, the dyadic constructs included in the review), which were further interpreted independently and qualitatively by researchers who are expert in the observation of mother-infant interactions. Whenever two researchers provided inconsistent interpretation, the lack of agreement was solved in conference with a third senior researcher (RM). The qualitative interpretation allowed to give sense to the quantitative information about the probability of two or more dyadic processes to be connected in time or as precursors or consequence of each other.

#### Methodological review

The included papers have been reviewed for methodological aspects including setting variables (e.g., laboratory vs. home environment), procedural variables (e.g., observational procedure, operational definition), and sample-related variables (e.g., sample size, sample socio-demographic and clinical characteristics). The selected variables are meant to provide a sufficiently complete picture of methods and procedures adopted in previous literature to measure mother-infant dyadic processes as well as to highlight the characteristics of the samples included in previous research. The methodological review was conducted only on papers for which the quality appraisal was found to be at least moderate (score < 3), whereas papers with quality of appraisal equal to 3 (poor) were excluded.

#### Systematic review of outcomes

First, the findings for the included papers have been reported without specific classifications, in order to avoid pre-oriented biased selection of outcomes by the authors. The methodological review was conducted only on papers for which the quality appraisal was found to be at least moderate (score < 3), whereas papers with quality of appraisal equal to 3 (poor) were excluded. When all the findings were included in an excel file, three independent authors (LG, GSdM, EG) provided a categorical label for each finding. Disagreement has been solved in conference with senior researcher LP. The final labels were: characterization, precursors, consequences, group-differences. Characterization includes findings related to the description of the selected dyadic process in terms of timing, actions and reciprocal contributions of the interactive partners. Precursors included individual, environmental or dyadic variables, which emerged as significant predictors of variations in the selected dyadic process. Consequences included the set of effects that the selected dyadic process was found to exert on cognitive, behavioral and affective development of infants and children. Group-differences included the outcomes of studies conducted on different mother-infant groups, generally representing healthy or low-risk populations compared to clinical or high-risk populations.

## Results

### Preliminary analysis

#### The “when,” “where,” and “who” of mother-infant dyadic processes research

**Figure 2:**
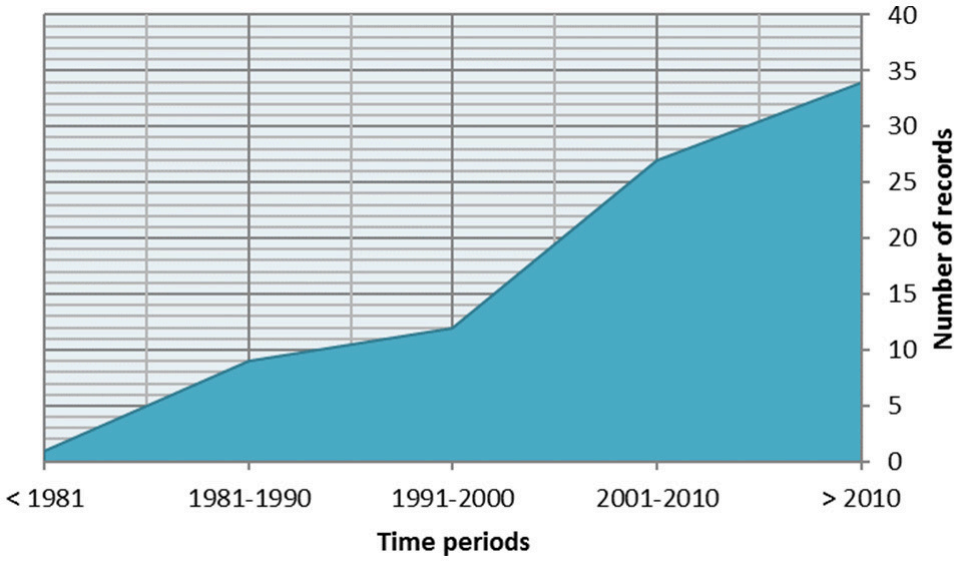
Number of retrieved records clustered in decades.

**Figure 3:**
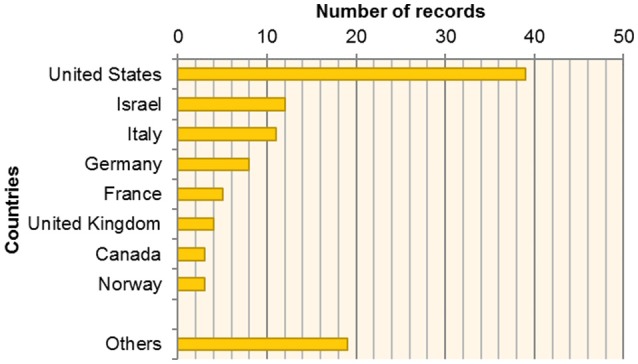
Distribution of retrieved records by country. “Others” includes papers from the following nations: Greece, Netherlands, Sweden, Belgium, Brazil, Denmark, Finland, Japan, South Africa.

A pool of 82 studies was finally included. The interest in the study of mother-infant dyadic processes has been growing during the last four decades (see Figure 2). Researchers from all over the world are interested in studying mother-infant dyadic processes, as illustrated in Figure 3.

#### Quality appraisal

The results of the quality appraisal of the included studies is reported in Table 2. The percentage of excluded studies due to poor quality appraisal ranged from 0% (Matching, 0/10 records; Mirroring, 0/3; Reciprocity, 0/5; Reparation, 0/4) to 66% (Mutuality, 2/3).

**Table 2:** Quality appraisal for the included studies.

**Dyadic concept/process**	**Study**	**Selection bias**	**Study design**	**Confounders**	**Blinding**	**Data collection**	**Withdrawals**	**Overall score**
**ATTUNEMENT**								
	Feldman and Greenbaum, 1997	2	2	2	2	1	3	2
	Jonsson et al., 2001	3	2	2	2	2	3	3
	Jonsson and Clinton, 2006	3	2	1	2	2	2	2
	Leyendecker et al., 1997	2	2	1	3	1	1	2
	Nicely et al., 1999b	2	2	2	3	2	1	2
**CONTINGENCY**								
	Beebe et al., 2010	2	2	1	2	1	2	1
	Beebe et al., 2011	2	2	1	2	1	2	1
	Beebe et al., 2012a	3	2	2	2	1	3	3
	Beebe et al., 2012b	3	2	2	2	1	3	3
	Beebe et al., 2016	1	2	1	2	1	3	2
	Bigelow and Rochat, 2006	2	2	1	2	1	1	1
	Braarud and Stormark, 2006	2	2	2	1	1	2	1
	Braarud and Stormark, 2008	1	2	2	1	1	1	1
	Brighi, 1997	3	2	2	2	1	2	2
	Cohn and Elmore, 1988	3	2	3	2	1	3	3
	Cote et al., 2008	2	2	1	3	1	3	3
	Crown et al., 1996	2	2	1	2	1	3	2
	Kärtner et al., 2010	2	2	1	2	1	1	1
	Lohaus et al., 2005	2	2	3	2	1	3	3
	Malatesta et al., 1989	2	2	1	2	1	1	1
	Mendes and Seidl-de-Moura, 2014	2	2	3	2	1	3	3
	Murray et al., 2016	2	2	2	2	1	3	2
	Pomerleau et al., 2003	2	1	1	2	2	3	2
	Striano et al., 2006	2	2	3	2	2	2	2
	Suwalsky et al., 2012	2	2	1	2	1	2	1
**COORDINATION**								
	Crown et al., 2002	2	2	1	3	1	2	2
	De Barbaro et al., 2013	3	3	3	3	3	1	3
	Feldstein et al., 1993	2	2	3	2	1	3	3
	Hammal et al., 2015	2	2	3	1	1	1	2
	Hane et al., 2003	2	2	1	2	1	1	1
	Harder et al., 2015	2	2	1	2	1	1	1
	Kokkinaki et al., 2017	2	2	1	2	2	2	1
	Montirosso et al., 2010	2	2	1	2	1	1	1
	Moore and Calkins, 2004	3	2	1	2	1	1	2
	Northrup and Iverson, 2015	2	2	1	2	2	1	1
	Provenzi et al., 2015b	2	2	3	2	1	2	2
	Rutter and Durkin, 1987	3	2	3	2	1	3	3
	Zlochower and Cohn, 1996	2	2	3	3	1	3	3
**MATCHING**								
	Deckner et al., 2003	2	2	2	2	2	3	2
	Field et al., 1990	2	2	1	2	1	3	2
	Montirosso et al., 2015	2	2	2	2	1	1	1
	Moore and Calkins, 2004	3	2	1	2	1	1	2
	Nicely et al., 1999a	2	2	2	3	2	1	2
	Nicely et al., 1999b	2	2	2	3	2	1	2
	Noe et al., 2015	2	2	2	1	1	3	2
	Reck et al., 2011	2	1	1	2	1	2	1
	Riva Crugnola et al., 2014	2	2	2	2	1	1	1
	Riva Crugnola et al., 2016	2	2	2	1	1	1	1
	Tronick and Cohn, 1989	2	1	2	2	1	2	1
	Weinberg et al., 2006	2	2	1	2	1	1	1
**MIRRORING**								
	Bigelow and Walden, 2009	2	2	1	2	1	1	1
	Kim et al., 2014	2	2	1	1	1	1	1
	Lavelli and Fogel, 2013	3	2	1	2	1	1	2
	Murray et al., 2016	2	2	2	2	1	3	2
	Sarfi et al., 2011	2	2	1	2	2	2	1
**MUTUALITY**								
	Savonlahti et al., 2005	3	2	1	3	1	3	3
	Van Egeren et al., 2001	2	2	3	3	3	3	3
	White-Traut et al., 2013	1	1	1	2	1	2	1
**RECIPROCITY**								
	Feldman, 2010	2	2	2	1	1	1	1
	Ferber and Feldman, 2005	2	2	3	2	1	1	2
	Lowinger, 1999	2	2	1	2	1	3	2
	Mayes et al., 1997	2	2	1	2	1	1	1
	Roe and Drivas, 1997	2	2	3	2	1	2	2
**REPARATION**								
	Montirosso et al., 2015	2	2	2	2	1	1	1
	Müller et al., 2015	2	2	2	1	1	2	1
	Provenzi et al., 2015b	2	2	1	2	1	3	2
	Reck et al., 2011	2	1	1	2	1	2	1
**SYNCHRONY**								
	Bernieri et al., 1988	3	3	3	2	3	2	3
	de Graag et al., 2012	2	2	1	3	2	3	3
	Doi et al., 2011	2	2	2	2	2	3	2
	Dowd and Tronick, 1986	3	3	2	3	2	1	3
	Feldman et al., 1996	2	2	1	2	1	1	1
	Feldman and Greenbaum, 1997	2	2	3	2	1	3	2
	Feldman, 2006	2	2	1	2	1	3	2
	Feldman et al., 2011	2	2	2	2	1	3	2
	Field et al., 1989	3	2	3	2	1	3	3
	Field et al., 1990	2	2	1	2	1	3	2
	Granat et al., 2017	3	2	1	2	1	2	2
	Gratier, 2003	3	2	2	2	2	3	3
	Ham and Tronick, 2009	3	2	3	1	1	2	2
	Hammal et al., 2015	2	1	3	1	1	1	2
	Harel et al., 2011	2	2	1	3	2	1	2
	Kaitz et al., 2010	3	2	2	2	1	1	2
	Karger, 1979	3	2	2	3	2	1	3
	Leclère et al., 2016	2	2	3	2	1	3	3
	Lester et al., 1985	2	2	2	2	1	1	2
	Lotzin et al., 2015	2	2	2	1	1	3	2
	Lotzin et al., 2016	3	2	2	2	1	3	3
	Moore and Calkins, 2004	3	2	1	2	1	1	2
	Moore et al., 2016	2	2	2	2	1	1	1
	Tronick and Cohn, 1989	2	1	2	2	1	2	1
	Van Puyvelde et al., 2010	3	2	2	2	2	3	3
	Weinberg et al., 2006	2	2	1	2	1	1	1

### Theoretical review

#### Theoretical definitions

In Table 3, theoretical definitions of the dyadic concepts and processes are reported. Notably, the definitions of mutuality and reciprocity did not include a processual characterization in terms of timing and rhythm. As such, they appeared to be broader concepts, rather than processes. These broader concepts might be considered as a meta-theoretical framework, which includes different dyadic processes that occur within the mother-infant interaction.

**Table 3:** Theoretical definitions and measurement of dyadic concepts and processes.

**Dyadic concepts**	**Theoretical definition**	**Measurement**
Mutuality	Mutual contribution of the interactive partners, which might not be equal in terms of frequency and intensity of the behaviors of the two partners.	N/A
Reciprocity	Reciprocal influence between interactive partners.	N/A
Attunement	Sharing of actions and intentions which includes maternal identification of infant's inner feelings/states and infant's comprehension that the mother is referring to his own original state.	Frequency of behavioral codes (*N* = 4)
Contingency	Reciprocal adjustment of trans-modal affective and behavioral signals within a micro-temporal window that leads to infants' learning and regulation skills and interactive patterns.	Qualitative global rates (*N* = 2) Frequency of behavioral codes (*N* = 3) Odds ratio/conditional probability (*N* = 3) Time series analysis (*N* = 3)
Coordination	Bidirectional rhythmic exchanges characterized by specific timing and turn taking which facilitates the reciprocal prediction of future behavioral states.	Correlation index (*N* = 1) Proportion of time (*N* = 1) Frequency if behavioral codes (*N* = 3)
Matching	Simultaneous exhibition of the same affective and/or behavioral state by the mother and the infant.	Amount of time (*N* = 1) Frequency of behavioral codes (*N* = 4) Proportion of time (*N* = 2) Qualitative global rates (*N* = 1) Latency to match (*N* = 1) Odds ratio/conditional probability (*N* = 1) Duration of behavioral state (*N* = 1)
Mirroring	Exaggerated/marked reflection of trans-modal child behaviors by the mother through imitation of affective quality reproduction in a temporally contingent way.	Frequency (*N* = 2)
Reparation	Dyadic process in which unmatched dyadic states are transformed in matched dyadic states producing opportunity to learn interactive strategies and to achieve better stress and emotion regulation.	Rate of reparation (*N* = 2) Latency to repair (*N* = 2)
Synchrony	Degree of congruence between trans-modal behaviors of two partners which is lagged in time and which promotes infants' learning of emotional regulation skills and the emergence of expectations on interactive repertoires.	Correlation index (*N* = 6) Frequency (*N* = 2) Proportion of time (*N* = 2) Shared-variance/Coherence analysis (*N* = 4) Odds ratio/conditional probability (*N* = 3)

#### Relationships among dyadic processes

**Figure 4:**
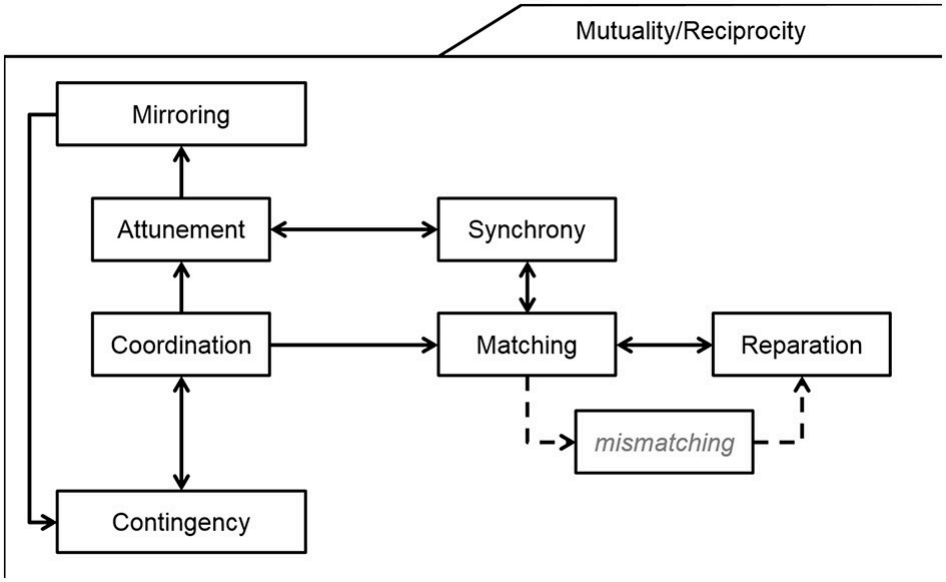
Theoretical model of the relationships among dyadic processes.

The computer-aided text analysis revealed a complex network of relationships among the included dyadic processes (Figure 4). Two dynamic cycles emerged.

First, the ability to share intentions (i.e., attunement)—rather than simple behaviors or actions within the dyad—emerged as a more complex mutual engagement between the mother and the infant which is built upon low-level contingent engagement (i.e., contingency and coordination). From this perspective, mirroring should be considered as a specific way of being together, which might only appear when the mother is able to understand the behavioral and inner states of the infant in order to provide an exaggerated version of the observed and inferred infants' socio-emotional state. When effective mirroring occurs, greater levels of contingency might be reached by the dyad, so that mother and infant constitute a dynamic system characterized by a behavioral-psychological self-organized and homeostatic cycle.

Second, a second cycle of matched and un-matched behavioral states within the dyad appeared to be regulated by dyadic reparation. Repeated matching emerged as the pre-condition for synchrony, which, in turn, contributed to heightened matching states. In other words, repeated in-moment matching states contribute to lagged moment-by-moment synchrony in time, so that reiterated interactive exchanges between mothers and infants grow in complexity in a reciprocal way.

In sum, as reported in Figure 4, coordination (of behaviors) and attunement (of intentions) might be considered as two critical nodes which allow the mother-infant dyad to move from behavioral forms of involvement (i.e., contingency, matching) to more complex psychological and inner-state forms of dyadic engagement (i.e., attunement, synchrony).

### Methodological review

#### Setting

Overall, about 70% of the studies was conducted in a lab environment. Free face-to-face interaction paradigms and the Face-to-Face Still-Face (FFSF) procedure were the most used experimental procedures (respectively, 46 and 30% of the entire pool of studies). Frequencies and percentages of setting variables (i.e., environment and observational procedure) are reported in Table 4, separately for each dyadic process.

**Table 4:** Setting of the included studies (percentages reported in brackets).

**Dyadic process**	**Environment**		**Observational procedure**					
	**Lab**	**Home**	**Free FTF interaction**	**Structured FTF interaction**	**Separation paradigm**	**Replay paradigm**	**FFSF procedure**	**Routine care**
Attunement	1 (20)	4 (80)	3 (60)	1 (20)	0 (0)	0 (0)	0 (0)	1 (20)
Contingency	13 (65)	7 (35)	12 (60)	3 (15)	1 (5)	3 (15)	0 (0)	1 (5)
Coordination	10 (77)	3 (23)	6 (46)	2 (15)	0 (0)	0 (0)	4 (31)	1 (8)
Matching	10 (83)	2 (17)	3 (25)	3 (25)	0 (0)	0 (0)	6 (50)	0 (0)
Mirroring	2 (50)	2 (50)	3 (75)	0 (0)	0 (0)	0 (0)	1 (25)	0 (0)
Reparation	4 (100)	0 (0)	0 (0)	0 (0)	0 (0)	0 (0)	4 (100)	0 (0)
Synchrony	21 (81)	6 (23)	13 (50)	1 (4)	0 (0)	0 (0)	10 (39)	2 (8)
Total	58 (72)	24 (30)	37 (46)	10 (12)	1 (1)	3 (4)	25 (30)	5 (6)

#### Sample

Sample characteristics are reported in Table 1, separated for each dyadic concept and process. Overall, a wide variation in sample size was observed, with a range of 14–133 (mean = 55.71, SD = 30.28). Greater sample sizes were reported in studies conducted on mother-infant contingency (mean = 70.10, SD = 34.94), whereas smaller sample sizes were included in studies on coordination (mean = 35.15, SD = 17.55). The mean age of infants ranged from 1 week to 36 months (mean age = 5.95 months, SD = 5.50). Younger infants were included in studies on mirroring (mean age = 2.86 months; SD = 2.20) whereas older children were included in studies on attunement (mean age = 7.13, SD = 4.85). The majority of included studies were conducted on healthy mother-infant dyads (*N* = 56), whereas at-risk or clinical condition were present in infants (preterm birth, siblings of children with autism, adopted infants, atypical development) and/or in mothers (depression, anxiety, minority status, low socio-economic status) of the remaining studies.

#### Measures

Table 3 reports information on the procedural methods adopted to measure each specific dyadic process. Wide differences exist in the ways the different dyadic processes have been measured and assessed in previous research. These methods range from simple counts of occurrences and measures of duration and latency to the adoption of correlation indexes, estimation of odds ratio, time-series and shared variance analyses. Notably, despite the fact that the theoretical definition of synchrony includes the lag-time criterion—which differentiates synchrony from matching, for instance—only a limited sub-group of synchrony-focused records included a defined lag (i.e., 4 out of 17 papers; 24%) or a non-constrained lag (i.e., 6 out of 17 papers; 35%) in which synchrony was measured. When specified a-priori, the lag varied between 1 (e.g., Feldman et al., 2011) and 3 s (e.g., Hammal et al., 2015). In 3 studies, the lag was set at 0, which is inconsistent with the theoretical assumptions of moment-by-moment synchrony adjustments.

### Systematic review of outcomes

#### Characterization of dyadic processes

##### Attunement

Mixed findings emerged for the relationship between mothers' attunement and infants' regulation. Mothers' attunement seems to be linked to six infant behaviors: pleasurable motoric behavior, effect initiation, focusing, loss of balance, uncontrolled behavior and displeasure (Jonsson et al., 2001). Moreover, a cross-age stability between maternal affect attunement and infant affect regulation at 3 and 9 months has been highlighted by Feldman and colleagues (Feldman and Greenbaum, 1997).

##### Contingency

The reciprocal adjustment of maternal and infant behavior is somehow asymmetric due to the fact that maternal contingent responses to infants' cues are more frequent than infants' contingent responses to maternal signals and behaviors (Beebe et al., 2016). On the other hand, altered patterns of maternal contingency has been associated with decreased infant direct speech and gaze (Braarud and Stormark, 2006, 2008; Striano et al., 2006) and the likelihood of being unable to detect contingent relations was higher for those children who are faced with low responsive and highly intrusive parental behaviors (Brighi, 1997). Moreover, infants tend to smile more contingently to their mothers' smiles than to strangers' smiles (Bigelow and Rochat, 2006). From a developmental point of view the infant's ability to be contingent appears early in life and increases particularly after 3 weeks (Crown et al., 1996; Murray et al., 2016).

##### Coordination

Mother-infant coordination has been considered from Kokkinaki et al. (2017) as an increase in the frequency of change in the mother's emotional state that is accompanied by an increase in the frequency of the infant's emotional changes and when mothers and infants displayed pleasure, interest, neutral, and negative emotions at the beginning of a sub-unit, they were more likely to express the same emotions at the end of a coherent sub-unit of engagement. A focal point of the dyadic coordination process concerns the role of timing in the interaction: interesting results come from the studies conducted by Crown (Crown et al., 2002) and Harder (Harder et al., 2015). They first observed a reciprocal coordination between infants and both mothers and strangers, and the magnitude of the coordinated interpersonal timing of pauses significantly differentiated the mothers and strangers when they were interacting with the infants. The proportion of time spent in coordinated vocal interaction significantly increased with age (Harder et al., 2015). At 4 months, it was 22% of the time, rising to 34% at 7 months. This proportion was stable from 7 to 10 months.

##### Matching

Matching has been found to increase with infants' age (from 3 to 6 and 9 months, Tronick and Cohn, 1989). Social engagement matching is generally more frequent than object engagement matching (Tronick and Cohn, 1989). Moore and Calkins (2004) reported similar levels of matching between Play and Reunion episodes of the Still-Face Procedure in healthy infants. Nonetheless, Weinberg et al. (2006) further documented that matching of negative affective states between mothers and infants was higher during the Reunion episode, probably reflecting the need of the mother to re-engage the infant back, after a stressful experimental condition.

##### Mirroring

Mirroring has been found to increase with age (from 3 to 9 weeks, Murray et al., 2016). Moreover, during the Still-Face Paradigm, mothers exhibit higher frequency of mirroring during the Play compared to the Reunion episode (Bigelow and Walden, 2009). Mirroring is also higher when the infants produce clear social signals and communications toward the mother (Lavelli and Fogel, 2013; Murray et al., 2016). Bigelow and Walden (2009) showed that maternal mirroring behaviors were in the same modality as the infants' behavior, which is indeed consistent with the theoretical definition of mirroring.

##### Reparation

Reparation has been observed in dyads of mother and 4-month-old infants, showing that greater reparation is observed in mother-infant dyads characterized by frequent social engagement (Montirosso et al., 2010).

##### Synchrony

Mother-infant synchrony has been observed as early as 3 and 5 months of life (Lester et al., 1985). Lower levels of synchrony are observed during Play compared to Reunion in the FFSF procedure (Moore and Calkins, 2004). During normal face-to-face interactions, synchrony and matching of affective states are highly correlated (Moore et al., 2016).

#### Antecedents of dyadic processes

##### Attunement

Leyendecker et al. (1997) observed how the securely attached dyads were significantly more likely than the insecurely attached dyads to be engaged in attuned interactions. Likewise, another study showed that affect attunement was often elicited by infant exploration and play (Jonsson and Clinton, 2006).

##### Contingency

N/A.

##### Coordination

Generally, the duration of the infants' latency to respond was a significant predictor of maternal latency in responding to infants' cues. Moreover, language delayed infants were more likely to speak when their mothers were vocalizing compared to the high risk and low risk non-delayed infants (Northrup and Iverson, 2015). Furthermore, comparing the FFSF Play and Reunion episodes, coordination of mothers and infants' head movement velocity was greater during Play (Hammal et al., 2015).

##### Matching

The amount of infants' positive emotionality displayed at 9 months has been documented to be predictive of the frequency of mother-infant matching at 13 months (Nicely et al., 1999b).

##### Mirroring

Lavelli and Fogel (2013) reported that the probability of the mother mirroring the infant's facial and vocal actions after infant's own social signals increased significantly during the first month of life. The reciprocal mirroring of infants in response to maternal communications showed the same developmental trajectory.

##### Reparation

N/A.

##### Synchrony

The role of infants' demographic variables in affecting mother-infant synchrony has received mixed evidence. On the one hand, a significant positive correlation between infants' age and synchrony has been observed by Doi et al. (2011), and remained significant even after controlling the influence of confounders. Similarly, Feldman et al. (2011) documented that synchrony increased from 3 to 9 months of infants' age. Tronick and Cohn (1989) showed that mother-son dyads had higher synchrony compared to mother-daughter dyads at 6 and 9 months. On the other hand, no significant effect of infants' age and sex emerged in other studies (Tronick and Cohn, 1989; Weinberg et al., 2006; Lotzin et al., 2016). The extent of infants' positive emotionality was not found to be a significant predictor of mother-infant synchrony (Weinberg et al., 2006). As for maternal antecedents of mother-infant synchrony, Moore et al. (2016) showed that maternal pre-partum anxiety was significantly correlated with lower mother-infant synchrony during the play episode of the FFSF procedure. Greater maternal dysregulation was associated with higher mother-infant gaze synchrony (Lotzin et al., 2015).

#### Consequences of dyadic processes

##### Attunement

Feldman and Greenbaum (1997) underlined that maternal affect attunement at 3 months correlated with Symbolic Play and Internal State Talk, while at 9 months it was correlated to Verbal IQ.

##### Contingency

In general, Malatesta et al. (1989) documented that maternal contingency predicted more infant gaze toward the caregiver and more frequent displays of positive facial emotion expressions by the infant. Pomerleau et al. (2003) found that the level of maternal contingency at 1 month significantly correlated with infants' performance on both mental and behavioral scales at age 6 months. The level of contingency observed within interactions of 4-month-old infants and their mothers was predictive of 12-month attachment classification (Beebe et al., 2010). Notably, secure attachment was predicted by a moderate level of contingency, whereas both high and low levels predicted insecure attachment classification. Mothers who displayed very low negative affective contingency had higher a probability of being mothers to an infant classified as disorganized in terms of attachment at 12 months.

##### Coordination

A significant curvilinear relation emerged between mother's coordinated interpersonal timing and coordination of non-interruptive simultaneous speech. Moreover, mothers who coordinated their non-interruptive simultaneous speech to that of their infants in moderate levels were characterized by high levels of overall sensitivity, and mothers highest in sensitivity were characterized by moderate levels of coordination (Hane et al., 2003).

##### Matching

Higher frequency of matching during the Play episode of the Still-Face Procedure has been associated with greater displays of positive emotionality by the infant during the Still-Face and the Reunion episodes (Noe et al., 2015). Specifically, infants of dyads characterized by high matching spent about twice as much time in positive emotionality states compared to infants of dyads characterized by low matching. Nicely et al. (1999a) showed that dyadic matching at 9 months was predictive of positive, but not negative infants' emotionality at the 13-month follow-up observation. Notably, mismatching was not a significant predictor of later negative emotionality of 13-month-old infants (Nicely et al., 1999b).

##### Mirroring

In a sample of mothers and 7-month-old infants, there was no significant predictive effect of dyadic mirroring (i.e., intention mirroring, as the authors define it) on the amount of time spent by the infant in looking toward or away from the mother (Kim et al., 2014).

##### Reparation

The latency to repair showed in mother-infant dyads at 3–4 months has been found to be significantly associated to infant cortisol reactivity and to significantly moderate the relationship between retrospectively reported emotional distress during the first trimester of pregnancy and cortisol-reactivity (Müller et al., 2015). Moreover, the rate at which mother-infant dyads repaired mismatch states during the Play episode of the Still-Face Procedure at 4 months was associated with infants' negative emotionality during the Still-Face and the Reunion episodes (Provenzi et al., 2015b).

##### Synchrony

The quality of mother-infant synchrony at both 3 and 9 months was found to be predictive of infant's symbolic play as well as the development of internal state talk (Feldman et al., 1996). Infants with less organized sleep cycles demonstrated less ability to participate in synchronous interactions with their mothers (Feldman, 2006). Mother-infant synchrony at 3 months was a significant predictor of visual Intelligent Quotient at 24 months (Feldman et al., 1996).

#### Group-differences in dyadic processes

##### Attunement

Comparing a sample of immigrants from Central America (CA) with people from middle-class Euro- American (EA) backgrounds, significant correlations between the percentages of time that attunement was scored and each functional context were found for the CA sample whereas only half of the correlations were significant in the EA sample.

##### Contingency

No significant differences emerged in the overall contingency level between cultural groups or with the infants' age from the study of Kärtner et al. (2010). On the other hand, Pomerleau (Pomerleau et al., 2003) found that high-risk and moderate-risk mothers were less contingent than low-risk ones. Comparing dyads by adoption status, vocal-attention interactions (speak-attend, attend-speak) were significantly contingent for both groups whether mothers or infants initiated them, and a similar contingency has been highlighted for infant-initiated vocal interactions (vocalize-speak) for both groups. Two pairs of behaviors were contingent for one group but not the other: attend-encourage (contingent for adoptive only) and vocalize-encourage (contingent for birth only) (Suwalsky et al., 2012).

##### Coordination

Comparing high risk and low risk non-delayed infants to infants with a language delay, latencies to respond appeared strongly and positively related to one other for high risk and low risk non delayed dyads, while the same pattern of coordination was not exhibited for language-delayed dyads (Northrup and Iverson, 2015).

##### Matching

Mixed findings emerged for gender. Higher levels of dyadic matching have been observed in dyads with males compared to dyads with female infants (Tronick and Cohn, 1989; Weinberg et al., 2006), whereas others found that dyads with females have higher mean odds ratios for matching (Deckner et al., 2003). Deckner also reported a significant increase in matching from 18 to 24 months in girls, but not in boys. As for maternal depression, Reck et al. (2011) showed that matching is significantly higher in dyads of non-depressed mothers. Similarly, Weinberg reported that moments of dyadic matching were more common in dyads of healthy mothers compared to dyads of depressed mothers. In another study on dyads with 3-month-old infants, maternal stress, anxiety and depression were moderately correlated with higher proportion of dyadic mismatched states (Riva Crugnola et al., 2016). Nonetheless, multiple regression analyses revealed that maternal anxiety alone was a greater predictor of mismatched states at 3 months, compared to depression and parental stress. Montirosso et al. (2010) examined dyads of preterm and full-term infants, documenting that preterm infants were more likely than full-term peers to use distancing strategies to cope with the maternal display of still-face. Moreover, regardless of birth status (i.e., preterm or full-term), the dyads showed less coordination and a slower reparation rate during the Reunion episode of the FFSF procedure compared to the Play episode. In Riva Crugnola et al. (2014), the duration of matched states was assessed in adolescent mothers compared to adult mothers of 3-month-old infants. More negative matched states were displayed by adolescent mothers' dyads, whereas more positive matching emerged in dyads of adult mothers. Moreover, dyads of mothers assessed as insecure at the Adult Attachment Interview showed less positive matched states compared to dyads of secure mothers and more frequent mismatches. This effect was more evident in dyads of adolescent mothers.

##### Mirroring

Kim et al. (2014) compared dyadic mirroring between dyads of 7-month-old infants and mothers with a secure or insecure/dismissing classification at the Adult Attachment Interview. Whereas no significant differences emerged for what the author call “direct mirroring,” which is much more similar to unilateral maternal imitation of infants facial and emotional displays, dyads of secure mothers engaged in intention (i.e., marked) mirroring more than twice than dyads of insecure/dismissing mothers.

##### Reparation

In dyads with depressed mothers, the rate of mismatched state reparation was lower compared to dyads with healthy mothers (Reck et al., 2011). Moreover, dyads with depressed mothers took significantly longer to repair mismatched states into positive matches, compared to healthy control dyads. Provenzi et al. (2015b) reported that dyads of infants who did not show adequate physiological regulation (i.e., absence of vagal tone suppression) during the Still-Face episode of the Still-Face Procedure showed fewer attempts of reparation during the Play episode, compared to dyads of infants who adequately suppressed vagal tone as a sign of good physiological stress regulation. Specifically, in terms of timing, in the non-suppressor group the average repair rate was approximately once every 10 s, whereas in the suppressor group it was approximately once every 6 s.

##### Synchrony

Dyads with preterm infants engaged in more frequent, but shorter episodes of mother-infant synchrony (Harel et al., 2011). Lester et al. (1985) documented higher synchrony in full-term dyads than in preterm dyads at 3 and 5 months. Feldman (2006) documented that synchrony was more prevalent in dyads of full-term compared to premature infant dyads. Dyads with depressed mothers showed less synchrony compared to dyads with non-depressed mothers (Field et al., 1990). Lower amount and latency to first synchrony occurrence emerged in dyads with depressed mothers compared dyads with non-depressed and anxious mothers (Granat et al., 2017). No differences emerged in the latency of the first synchronous state between dyads with anxious and non-anxious mothers (Kaitz et al., 2010).

## Discussion

The present work represents an attempt to provide a first comprehensive, integrative and multi-dimensional account of more than four decades of research on mother-infant dyadic processes. Our attention has been directed to three main levels of analysis—theory, methodology and findings—which will be described in the following paragraphs.

Nonetheless, preliminary findings also provided important information suggesting that the interest in the study of dyadic processes is still growing, and during the current decade (i.e., 2010s), for the first time, the number of published articles on this topic is higher than 30. Moreover, the geography of this research area suggests that the main contribution are USA-based (featuring the seminal works of authors such as Beatrice Beebe and Ed Tronick), despite non-negligible contributions have also been provided by Ruth Feldman (Israel) as well as researchers based in Italy, Germany and France. Notably, there is a lack of research in non-Western cultures on mother-infant interaction. Despite the fact that the exploration of cultural influences on mother-infant dyadic processes is not a focus of the present work, we would like to highlight that this might be one of the more promising directions of future research. Finally, the top journals publishing papers on dyadic processes to date include Infant Behavior and Development, Developmental Psychology, and Infancy (more than five records each).

### Depicting the complex landscape of dyadic concepts and processes

We reported the definition of the different dyadic constructs in Table 3. First, it is immediately clear that two (i.e., mutuality and reciprocity) among the nine constructs abstracted here seem to be much better framed as wide, global and meta-theoretical concepts rather than as processes. Both these concepts provide a specific point of view on mother-infant interaction, assuming that there are reciprocal or mutual contributions within the “dyadic dance.” Nonetheless, they are different in the way they underline the relative contributions of each of the interactive patterns. Indeed, reciprocity assumes that the contribution of the mother and the infant are in some way equal in terms of relevance, frequency and intensity (Trevarthen, 1980), whereas mutuality may represent a more cautious and conservative approach in which both partners contribute to the dyadic system, but with different quantities and qualities (Beebe et al., 2010). The first approach appears to be more conducible to the first contributions from Trevarthen and Stern, whereas the mutual model is more consistent with the proposals of Beebe and Tronick.

The other seven constructs appear to be real dyadic processes, with both theoretical and methodological definitions. These processes appear to be in specific relationships among themselves. First, contingency emerges as the low-level process, in which the dyadic encounter is expressed by the concomitant expression of specific gestures. When these contingent moments repeat in time they generate more prolonged states of coordination, which require not only an in-moment contingency but a sequence of non-necessarily continuous contingent moments organized in a stable pattern in space and time. Coordination appears to be a critical node that leads on the one hand to attunement and mirroring and on the other to the matching-reparation cycle and to synchrony.

This latter cycle is well known and represent the core theoretical proposal made by Tronick (e.g., Tronick and Beeghly, 2011). It means that the matching state is not meant to be the stable nor the all-time desirable state of the dyad; instead, continuous interactive ruptures and re-negotiations occur within the dyad which alternatively experience states of matching, ruptures, reparation, and matching again. Tronick has greatly described how the ability to repair interactive ruptures is one of the central dyadic processes that associates with adequate and protective caregiving (DiCorcia and Tronick, 2011) as well as effective psychotherapeutic work (Harrison and Tronick, 2011; Fonagy, 2015). Based on the present work, synchrony appears to be a higher level process in which not only the dyad navigates the process of moment-by-moment negotiation of dyadic states, but also does that in a common and joint way. Again, synchrony is also meant to be a higher level process as it is critically trans-modal: whereas matching is generally described as the simultaneous display of similar affective states (e.g. mother positive and infant positive), synchrony is not limited to perfect matches, but rather to smooth on-line changes in the agenda of each of the interactive partners.

The other cycle regards the vertical emanation of coordination reported in Figure 4. When coordination is achieved in the dyad, it is also possible to coordinate not only behavioral outputs, but also intentions and affects. This is what Stern meant by the use of the word “attunement,” referring both to the mother-infant dyad and to adult lovers. The attunement of intentions and affects also makes it easier for the member of the dyad—more often the mother—to understand what is going on between them, to anticipate the other subject's act on the basis of this intention forecasting and to mirror the reciprocal expression with exaggerations that hold the meaning: “I understand exactly how you feel.” Finally, the ability of being in “togetherness” states characterized by high levels of attunement and mirroring is also a facilitating dyadic context for the emergence of new contingent states, which makes this set of relationship a cycle which repeats in time and space.

### Milestones of mother-infant dyadic processes research

Even though mother-infant interaction is one of the most natural processes of our life, the majority (approximately 2 out of 3) of the included studies reported lab-based researches. The most used settings include free face-to-face interactions and the stress-inducing FFSF procedure. Importantly, these processes have been observed ubiquitously at different ages, ranging from the very first week to the third year of life. Consistently with the model reported above in which attunement is meant to be one of the highest level process, this has been more studied in older children. Moreover, as mirroring is more often displayed by the mother who anticipate infants' intention by providing a meaning-making context for his/her communicative signals this has been observed even in younger infants.

The great majority of this research also focused on healthy mother-infant dyads, in which neither the infant nor the mother presented clinical complications or risk factors. Nonetheless, there is a notable proportion of studies which reported how dyadic processes occur when the infants' present specific clinical conditions such as preterm birth or risk-factors such as being a sibling of a child diagnosed with autism spectrum disorder or when the mothers present signs of psychological disturbances, including depression, anxiety, and stress.

As expected, different methods emerged for each of the seven dyadic processes abstracted here. For some of the processes (i.e., attunement and mirroring) only one measure has been reported, i.e., frequency of the specific dyadic behavioral coding. Reparation only reported two methods as well, which included the number of repairs made by the dyad and the latency to the first repair, especially when it was measured in association with the FFSF procedure. Other processes featured three or more measures, such as synchrony (correlational indexes, frequency, proportions of time, shared variance, odds ratio and conditional probability), contingency (qualitative global rates, frequencies, time series analysis, odds ratio and conditional probability), coordination (correlational indexes, proportion of time, frequencies), and matching (raw amount of time, frequencies, proportion of time, qualitative global rates, latency to match, duration of behavioral state, and odds ratio and conditional probability). Despite the majority of these measures were based on the fine-tuned and frame-by-frame or second-by-second micro-analytical coding of videotaped mother-infant interactions, qualitative/macro-analytical account of specific processes (e.g., matching) also exist. Nonetheless, as previously suggested (Cerezo et al., 2012), the micro-analytical account appears to be more consistent with the theoretical and meta-theoretical framework of the non-linear dynamic systems, despite it is more expensive in terms of human resources and time.

### Why do dyadic processes matter?

Research on the antecedents of dyadic processes have poorly focused on contingency and reparation. Whereas this is less surprising for contingency—as it is a very low complexity-level dyadic process—it is really unfortunate that previous studies did not provide specific information for what pertains which are the main factors associated with more effective and protective reparation processes within the mother-infant dyad during normal (Riva Crugnola et al., 2013) and stressful (Tronick, 2007) face-to-face interactions. As Tronick (2007) intended dyadic reparation as a key element of the dyadic functioning which has direct implications for behavioral and emotional health and well-being, future research is meant to dedicate specific efforts in this direction. As for the other processes, generally there is evidence that both infants' and maternal behavioral characteristics are factors associated with the display of more adaptive dyadic processes. For example, infants at different ages who play more, display more positive emotionality and freely explore the environment also experience dyads in which the amount of attunement, coordination, matching and synchrony is higher. The mother affects the quality of the dyadic encounter, as those more securely attached show more attunement and those with higher prenatal anxiety are less able to engage in synchronous interactions.

Research on the consequences of dyadic processes for the behavioral, cognitive and emotional development of infants have provided relevant information. First, the majority of the processes investigated here have multiple effects on infants' growth and development, including both cognitive (e.g., IQ is higher in infants who experienced more attuned and synchronous interactions with the mother), behavioral (e.g., greater matching is predictive of more positive and stable behavior in infants), affective (e.g., more contingency is associated with greater probability to develop a secure attachment), and biological markers of stress regulation (e.g., better reparation rates are associated with less pronounced cortisol reactivity to the FFSF procedure). In sum, all the processes have been shown to have protective and beneficial effects on both healthy and at-risk infants. Unfortunately, very little is known about the effects of engaging in dyadic interactions on maternal health and well-being. In the light of the mutuality and reciprocity features of the different theoretical models of mother-infant dyadic functioning, the lack of studying the consequences of mother-infant dyadic processes on maternal behavioral and emotional states is surprising and surely deserves more attention in future research.

### Open questions and future directions

This review aimed at a first attempt to organize terms, methods and knowledge on caregiver-infant research. Beyond this initial systemization, the research area is still confronted with several uncertainties due to gaps in the empirical level. First, the majority of studies on caregiver-infant systems were implemented in western societies (see Figure 3). Cross-cultural comparisons to uncover society-specific characteristics in caregiver-infant processes, thus, are impossible and consequently one major challenge of future research. Moreover and not less vital, some interactional formats have widely been neglected in caregiver-infant research. On the one hand, most of our knowledge on caregiver-infant dyads derives from studies that observe interaction processes between mothers and their infants. There are only a few studies on father-infant dyads: For example, Feldman (2003) found indications that gender differences regarding synchrony may refer to gender-specific patterns of arousal and depend on the matching between the infant and parental gender in the observed dyads. Thus, more comprehensive studies on paternal engagement and the dyadic processes in father-infant dyads would extend existing knowledge on caregiver-infant systems. On the other hand, there are very few studies on interaction processes in triadic settings.

Second, the model reported in Figure 4 is the product of a theoretical attempt of integration. Future research may use this concept as a starting point for targeted and direct examinations of reciprocal interconnections between the dyadic processes that were the focus of this framework. In other words, this model might be considered as an *a priori* model to be tested with original data in different populations of infants and mothers. Additionally, the suggested existence of developmental steps from contingency to mirroring warrants further investigation in infants with different ages or risk-status. In this context, little is known about the developmental antecedents of dyadic reparation processes. Thus, parental and infant characteristics that determine the reparation process need to be addressed in future investigations, especially as they represent a main feature for healthy infant development (DiCorcia and Tronick, 2011).

Third, a comprehensive systemization of the complex processes involved in caregiver-infant-dyads might lead to new insights for early therapeutic interventions that aim at transforming maladaptive interaction patterns into adaptive ones (Reck et al., 2004; Downing et al., 2014). Moreover, very little information was gathered about the effects of adaptive dyadic engagement patterns on the behavioral and emotional states as well as on long-term well-being of the caregivers. For the purpose of therapeutic approaches regarding parental mental health, this lack of knowledge has to be compensated.

Fourth, the great majority of research on dyadic processes have been conducted in laboratory setting (70% of studies). Despite many of these studies reported on well-validated and standardized procedures (e.g., the FFSF), interactions occurring in the laboratory environment may partially overlap with observations in more naturalistic contexts. More research is needed on dyadic processes in daily and home environments and to provide methodological relevant insights on the comparison of dyadic functioning in daily vs. novel environments. This kind of research would add to generalizability of the findings.

Fifth, the study of the contribution of individual dimensions (e.g. infant characteristics, such as gender: Weinberg et al., 2006; maternal characteristics, such as attachment classification: Riva Crugnola et al., 2014) appear to be under-represented among the pool of papers included in the present review. Nonetheless, the investigation of how individual dimensions contribute to the emerging dyadic processes that characterize the dyadic system is warranted to be further pursued in future research, as this field of studies may provide relevant insights on the protective factors that parents and infants bring into the dyadic functioning.

Last but not least, more research on the interconnections between macro-analytical concepts in caregiver-infant research, such as sensitivity and attachment, and micro-analytical processes is desirable. For instance, it was revealed that secure attachment may lead to more adaptive dyadic processes (Leyendecker et al., 1997) and that more adaptive dyadic processes are key to develop secure attachment (Beebe et al., 2010). Thus, the evolvement of secure attachment and adaptive interaction patterns may represent a dynamic auto-enforcing process in itself. Consistently, future investigations on the relations between macro- and micro-analytical concepts would not only connect different methodological approaches but also enhance our understanding of the dynamics in developmental trajectories.

## Conclusions

More than 40 years of infant research have completely revolutionized the scientific view of the mother-infant dyad. Laboratory and clinical research has provided new insights on the active role of the infant, and on the reciprocal and mutual contributions that both mothers and infants give within the dyadic encounter and the relevant impact of these precocious interactions for the long-term programming of infants' behavioral, cognitive, and socio-emotional development. To the best of our knowledge, this work is the first attempt to conceptualize a comprehensive systemization on this topic. Understandably, the development of concepts and methods in this area of research has not reached an end. Consistently, this work does not have an exhaustive purpose in itself, rather it merely holds the potential to better organize and orient future research in the field.

First, the preliminary analyses of this study highlighted the scarcity of mother-infant interaction research in non-western countries, which therefore needs to be endorsed in order to have a more global picture of the similarities and differences that could occur. Indeed, it would be interesting to investigate if the meta-theoretical model that we proposed (see Figure 4) holds well in these cultures as well, or rather if different interactive patterns take place.

Second, while there are some constructs that have been analyzed in detail (i.e., contingency, coordination, matching, synchrony), there are still some areas that have not been fully and thoroughly explored and deserve further attention (i.e., reparation and mirroring). It should be noted that these two processes are key dyadic mechanisms respectively involved in achieving better synchrony (i.e., reparation) and in increasing the chances of understanding the other partner's interactive intentions (i.e., mirroring). From this perspective, future studies should focus on exploring the relationship between mother-infant reparation and dyadic synchrony in healthy and at-risk conditions as well as on the role of mirroring in sustaining the development of social cognition in infants and children.

Furthermore, the knowledge on the processes that take place within mother-infant interaction potentially holds implications for clinical practice. Despite family-centered interventions for infants at developmental risk have been widely informed by the infant research field, the present review provides specific insights to aspects of the interactive exchange that should be targeted to support better infant-caregiver relationship and therefore sustain children's development. For example, in infants with severe neurodevelopmental disabilities contingency appears to be the most suitable and pursuable aim, whereas in older children with behavioral and conduct problems the focus might be on higher-order dyadic processes (e.g., attunement, synchrony and mirroring). In other words, we believe that a comprehensive knowledge of the mother-infant dyadic dance in its interactive components might increase our ability to take care of infants and children who present developmental risk.

## Author contributions

LP conceived the study, contributed to data synthesis and wrote the initial draft. LG and EG contributed to literature search, data abstracting and analysis. MM contributed to data abstracting and writing. GSdM contributed to literature search, data abstracting, analysis and English-language check. All the authors approved the final draft of the manuscript.

### Conflict of interest statement

The authors declare that the research was conducted in the absence of any commercial or financial relationships that could be construed as a potential conflict of interest.
